# Psychological Flexibility and Its Relationship to Distress and Work Engagement Among Intensive Care Medical Staff

**DOI:** 10.3389/fpsyg.2020.603986

**Published:** 2020-11-04

**Authors:** Johan Holmberg, Mike K. Kemani, Linda Holmström, Lars-Göran Öst, Rikard K. Wicksell

**Affiliations:** ^1^Centre for Psychiatry Research, Department of Clinical Neuroscience, Karolinska Institutet & Stockholm Health Care Services, Stockholm, Sweden; ^2^Department of Clinical Neuroscience, Karolinska Institutet, Stockholm, Sweden; ^3^Stress Research Institute, Stockholm University, Stockholm, Sweden; ^4^Department of Psychology, Stockholm University, Stockholm, Sweden

**Keywords:** intensive care, psychological flexibility, perceived stress, work engagement, occupational health

## Abstract

Intensive care settings place specific work-related demands on health care professionals that may elicit stress and negatively influence occupational health and work engagement. Psychological flexibility has emerged as a promising construct that could help explain variation in reported health. Understanding the role of psychological flexibility in occupational psychological health among intensive care medical staff may potentially guide the development of effective interventions. Thus, the present study evaluated the relationships between psychological flexibility (Work-related Acceptance and Action Questionnaire), distress (Perceived Stress Scale, General Health Questionnaire) and work engagement (Utrecht Work Engagement Scale) in a sample of 144 health care professionals from one adult (ICU, *N* = 98) and one pediatric (PICU, *N* = 46) intensive care unit. In addition to cross-sectional analyses, a subset of data (PICU, *N* = 46) was analyzed using a longitudinal design. Results illustrated that higher levels of distress were associated with lower levels of work engagement. Furthermore, psychological flexibility was related to greater work engagement, and psychological flexibility had a significant indirect effect on the relationship between distress and work engagement. Lastly, increased psychological flexibility over time corresponded with increased work engagement. Although tentative, the results suggest the importance of psychological flexibility for work engagement in health care professionals within intensive care settings.

## Introduction

Stressful situations are a well-known ingredient of the work context for health care professionals in intensive care settings. Existing research illustrates several specific antecedents eliciting stress among staff, e.g., workload, interpersonal conflicts (patients, families, and teams), moral distress, and issues of life and death (Crickmore, 1987; Embriaco et al., 2007; van Mol et al., 2015; Pereira et al., 2016; Elshaer et al., 2018; Kwiatosz-Muc et al., 2018). Furthermore, these factors have been shown to be associated with consequences in work-related outcomes, such as intention to leave (Khan et al., 2019), turnover (Adriaenssens et al., 2015), and work performance (van Mol et al., 2015), and health related outcomes, including burnout, traumatic stress, depression, and fatigue (Mealer et al., 2009; van Mol et al., 2015). Hence, existing studies have shown an elevated prevalence of distress among intensive care medical staff (Mealer et al., 2009; van Mol et al., 2015). Notably, considerable differences in levels of distress reported at different intensive care units indicate a need for further analysis, and in a systematic review including 40 studies van Mol et al. (2015) report a range of 0 to 70.1% in self-reported burnout assessed by Maslach Burnout Inventory (MBI). In addition to variation between units, there are also large variations in reported distress within units, also between health care staff working in the same positions who were exposed to similar stressors (McVicar, 2003; van Mol et al., 2015).

The large variation in levels of stress reported by health care staff may partially be explained by individual psychological qualities, including self-efficacy, personality, coping skills, and motivation. *Psychological flexibility* is a construct developed within the tradition of contextual behavioral science and can be defined as an ability to act in accordance with goals and values also in the presence of interfering psychological experiences (Hayes et al., 1999; Hayes et al., 2005). Thus, psychological flexibility does not primarily concern the presence of symptoms of distress, but rather the individual’s resilience and ability to function well in the presence of distress. It is the target of treatment in Acceptance and Commitment Therapy (ACT; Hayes et al., 2005), and measures of psychological flexibility have been shown to mediate outcomes in clinical trials in a variety of conditions such as chronic pain and anxiety (Hayes et al., 2013; Stockton et al., 2019). It has been argued that psychological flexibility plays a significant role in psychological health (Kashdan and Rottenberg, 2010) and studies have shown an incremental utility of psychological flexibility over traditional measures of distress (Gloster et al., 2011). However, more research is needed, including studies that evaluate the correspondence between psychological flexibility and general distress, as well as assessment instruments’ ability to discriminate between the constructs (Wolgast, 2014; Rochefort et al., 2018).

Several studies have evaluated the efficacy of ACT-based interventions within occupational health, and a meta-analysis by Öst (2014) conclude it to be possibly efficacious in targeting stress. Results from these studies also suggest that psychological flexibility may mediate changes in outcome (e.g., Flaxman and Bond, 2010; Brinkborg et al., 2011; Lloyd et al., 2013).

Valid measures of work-related psychological flexibility in health care settings enable research and development of interventions. Recently, the Work-related Acceptance and Action Questionnaire (Bond et al., 2013) has been validated in health care professionals (Xu et al., 2018; Holmberg et al., 2019). Data from these cross-sectional studies have shown higher levels of psychological flexibility to be associated with lower levels of psychological distress (e.g., stress, general psychological health, neuroticism, emotional exhaustion, and cynicism) and higher levels of workplace functioning (e.g., professional efficacy, task performance, job satisfaction, and work engagement) (Xu et al., 2018; Holmberg et al., 2019).

Psychological flexibility can be seen as a *resilience* factor. Resilience has commonly been defined as overcoming adversity or “effective functioning, despite the exposure to stressful circumstances, and/or internal distress” (Sturgeon and Zautra, 2013), and has gained increased attention and empirical support within occupational health and health care (Mealer et al., 2012; Yu et al., 2019). In a systematic review, Yu et al. (2019) conclude that despite lack of consensus, nurse resilience is an important construct that has received considerable attention, and there is a need to better understand factors contributing to nurse resilience. Recently, resilience was discussed within a contextual behavioral framework, and defined as “the ability to continuously engage in meaningful activities that promote current and future quality of life and health, in the presence of pain and distress” (Goubert and Trompetter, 2017). Thus, in addition to explaining variation in health and functioning, resilience is a potential target for behavioral health interventions. Also, conceptual similarities imply that psychological flexibility may be seen as a resilience factor.

Stress is a well-known aspect of occupational health, but the predominance of focus on ill-being in research has also been challenged (Myers, 2000; Schaufeli and Salanova, 2011; Jarden et al., 2018); attending to other aspects of health could improve interventions within occupational health. This has partly been illustrated during the last decades by research on *work engagement* (Bakker and Demerouti, 2008), defined as a “positive, fulfilling, work-related state characterized by vigor, dedication, and absorption.” (Schaufeli et al., 2002, p. 74). Publications on work engagement, particularly in nursing, have increased the last decade resulting in several recent systematic reviews (e.g., Keyko et al., 2016; Knight et al., 2017; Knight et al., 2019; Lesener et al., 2019). Results from these studies show higher levels of work engagement to be associated with positive outcomes, e.g., higher levels of job performance (Keyko et al., 2016), lower levels of intention to leave (De Simone et al., 2018; Wan et al., 2018), and higher levels of perceived work ability (Tomietto et al., 2018). There is yet a scarcity of studies exploring the relationship between psychological flexibility and work engagement among health care professionals (Xu et al., 2018; Holmberg et al., 2019; Solms et al., 2019) and more research is needed to clarify the roles and utility of these constructs.

To summarize, elevated distress is seen among health care professionals, particularly within intensive care settings, with specific work-related antecedents and negative effects on health and performance. A growing body of evidence points at the importance of work engagement for work-related outcomes. Psychological flexibility as a resilience factor may be an important and addressable factor to improve health and work performance in intensive care settings, but more research is needed.

Thus, the aim of this study was to explore the relationship between psychological flexibility, distress (perceived stress, general mental health), and work engagement among health care professionals within intensive care. More specifically, the following research questions were addressed:

(1) What is the strength and direction of the relationships between psychological flexibility, distress, and work engagement?
(2) Does psychological flexibility explain a significant amount of variance in work engagement, with and without controlling for distress?
(3) Is there an indirect effect of psychological flexibility on the relationship between distress and work engagement?
(4) Is increased psychological flexibility associated with increased work engagement over time?

## Materials and Methods

### Design

To explore the role of psychological flexibility in relation to work engagement and distress among intensive care staff, the present study utilized a cross-sectional design with additional longitudinal analyses for a subsample of participants (PICU).

### Procedure

Data was collected using the following self-report questionnaires: The Work-related Acceptance and Action Questionnaire (WAAQ), the Perceived Stress Scale-10 (PSS-10), the General Health Questionnaire-12 (GHQ-12), and the Utrecht Work Engagement Scale-17 (UWES). Background variables included profession, age, years of work experience and gender. In the longitudinal design data were collected at three separate occasions with approximately 1 month between each assessment.

All participants completed the informed consent form, containing information regarding the study, collection and storage of data, and the possibility to accept or decline participation. The study was approved by the local ethical review board in Stockholm, Sweden (Registration number 2014/42-31/3 and 2015/1881-32/3).

### Participants

Participants were recruited during staff training provided to two ICU’s, one adult ICU-unit and one pediatric ICU-unit, located at two different Swedish hospitals. Total number of staff participating at lectures were 124 from the ICU and 105 from the PICU. Response rates of staff choosing to participate in the study were 78% (98 out of 124) from ICU and 44% (46 out of 105) from PICU. Thus, the total sample consisted of 144 (98 + 46) health care professionals, including 58% nurses, 33% assistant nurses, 6% physicians, and 3% miscellaneous (manager, counselor). Mean age of the total sample was 46.6 (SD = 10.2) years, which consisted of 129 women and 14 men (one missing value) with a mean work experience of 19.6 (SD = 11.6) years.

### Self-Report Questionnaires

*The Work-related Acceptance and Action Questionnaire* (WAAQ; Bond et al., 2013) is a measure of psychological flexibility in occupational settings, including seven items rated on a Likert scale from 1 (Never true) to 7 (Always true), e.g., “I can admit to my mistakes at work and still be successful,” and “I can work effectively, even when I doubt myself.” WAAQ has a total score of 7 to 49, with higher scores indicating higher levels of psychological flexibility). WAAQ has been translated to Swedish and validated in a sample of health care professionals (Holmberg et al., 2019), showing good internal consistency (Cronbach’s alpha 0.85) and test-retest reliability (ICC 0.85). Cronbach’s alpha in the present sample was 0.87.

*The Perceived Stress Scale-10* (PSS-10; Cohen et al., 1983) was developed to assess cognitive appraisal of stress, i.e., to what extent situations are perceived as stressful. Examples of items are: “In the last month, how often have you felt that you could not cope with all the things you had to do?”, and “In the last month, how often have you felt that you were on top of things?”. Items are rated on a Likert scale from never (0) to very often (4). The total score ranges from 0 to 40 with higher scores indicating greater levels of perceived stress. PSS is validated in Swedish (Nordin and Nordin, 2013) on a sample from the general population and showed a Cronbach’s alpha of 0.84. Cronbach’s alpha in the present sample was 0.85.

*The General Health Questionnaire-12* (GHQ-12; Goldberg and Hillier, 1979; Goldberg et al., 1997) was originally developed to detect psychiatric illness, primarily depression. GHQ-12 has since then also been used as a general measure of mental health and psychological distress. Twelve items that reflect different aspects of health are rated on a Likert scale from 0 (strongly agree) to 3 (strongly disagree). Examples of specific items are “I have recently lost much sleep over worry” and “I have been thinking of myself as a worthless person.” Different methods of scoring exist. The preferred method to use for comparison between groups, and the one used in this study, is the Likert method which provides a total score of 0 to 36. Higher scores indicated higher levels of distress. A study validating the Swedish version of the GHQ-12 in a general population reported a Cronbach’s alpha of 0.83–0.89 (depending on scoring method) (Lundin et al., 2016). Cronbach’s alpha in the present sample was 0.75.

*The Utrecht Work Engagement Scale* (UWES; Schaufeli and Bakker, 2004). UWES measures *work engagement*, defined as “a positive, fulfilling, work-related state characterized by vigor, dedication, and absorption” (Schaufeli et al., 2002, pp. 74). Examples of items are “I’m enthusiastic about my job,” and “When I get up in the morning, I feel like going to work.” UWES consists of 17 items rated on a Likert scale from 0 (never) to 6 (always/every day). The total score comprises the mean of the 17 items, with higher scores indicating higher levels of work engagement. Results from previous studies have suggested two possible factor solutions, with either one (work engagement) or three factors (vigor, dedication, and absorption) (Schaufeli and Bakker, 2004). A Swedish version of the UWES was validated in a sample of information communication technology consultants by Hallberg and Schaufeli (2006), who found the one-dimensional and three-dimensional representations of work engagement to be equivalent with adequate fit measures for both. Cronbach’s alpha was 0.93 for the total score, 0.85 for vigor, 0.89 for dedication, and 0.76 for absorption. Cronbach’s alpha in the present sample was 0.90 for the total score, 0.78 for vigor, 0.86 for dedication, and 0.74 for absorption.

### Statistical Analyses

All analyses were calculated with the software IBM SPSS statistics, Version 26. The mediation analyses were calculated using the PROCESS plugin for SPSS (Hayes, 2018).

Bivariate analyses were used to broadly characterize the relationships between variables (i.e., strength and direction). Since not all variables were normally distributed as assessed by Shapiro-Wilk’s test (*p* < 0.05), Spearman’s rank-order correlation was used. The monotonic relationship between variables required by Spearman’s rank order correlation was determined by observation of scatter plots of related variables (Shapiro and Wilk, 1965; Razali and Wah, 2011).

Hierarchical regression analyses were used to further evaluate the correspondence between variables by assessing the amount of variance in UWES explained by WAAQ, both with and without controlling for distress (as assessed with PSS-10 and GHQ-12). All self-report questionnaires were treated as continuous variables. The linear relationship between dependent and independent variables was determined by visual inspection of scatterplots and partial regression plots. Independence of observations was determined by Durbin-Watson statistics. Homoscedasticity of residuals was examined by visual inspection of a plot of the studentized residuals and unstandardized predicted values. Outliers of standardized residuals larger than ± 3 SD were removed. Additional unusual points were assessed based on their influence on the models, such as high leverage points above 0.2 and highly influential points, as measured by a Cook’s distance above 1 (Cook and Weisberg, 1982).

**FIGURE 1:**
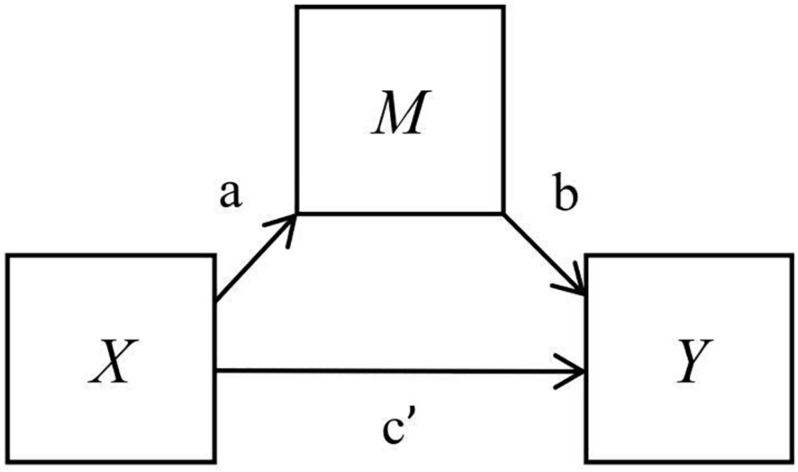
A simple mediation model showing the indirect effect of *M* on the relationship between *X* and *Y* (ab), and the direct effect between *X* and *Y* (c’).

Two analyses of indirect effects were conducted on the cross-sectional dataset; the indirect effect of WAAQ (*M*) on the relationship between PSS-10 (*X*) and UWES (*Y*), and the indirect effect of WAAQ (*M*) on the relationship between GHQ-12 (*X*) and UWES (*Y*) (see Figure 1). The PROCESS plugin for SPSS was used to assess the strength of the indirect effects (Hayes, 2018). In PROCESS, total, direct and indirect effects are calculated and tested for significance, as well as the respective paths, i.e., the *X* to *M* relation (*a* path), and *M* to *Y* relation (*b* path) (MacKinnon et al., 2007; Preacher and Hayes, 2008; MacKinnon and Fairchild, 2009). The mean value for the *ab* (*a* × *b*) product across the bootstrapped samples provides a point estimate of the indirect effect. Confidence intervals (CI) are derived from the obtained distribution of *ab* scores, using a 95% CI level which represents a significance level of *p* < 0.05. If lower and upper bounds do not contain zero, the indirect effect is significant (at the level specified in the analysis). Each analysis was based on 5000 bootstrapped samples, as suggested by Preacher and Hayes (2008).

In addition, we evaluated potential changes in work engagement (UWES) over time and the correspondence of these prospective changes with potential changes in psychological flexibility (WAAQ) and perceived stress (PSS-10). Included time-varying covariates (WAAQ; PSS-10) were grand mean centered to facilitate interpretation of their possible contribution to the model (Bolger and Laurenceau, 2013). Data based on three assessment points from a subsample of participants (PICU) was analyzed using Linear Mixed Models (LMM). Random effects and their associated covariances were retained based on their model contribution, as determined by model comparisons of Akaike’s Information Criterion (AIC; Burnham and Anderson, 2004). Under the assumption that data were missing at random (MAR), Restricted Maximum Likelihood (REML) estimation was used to model parameters and standard errors, based on all participants who provided at least one valid assessment for the dependent variables (Schafer and Graham, 2002). The assumptions pertaining to the normal distribution of residuals and homogeneity of variance were assessed, respectively, based on visual evaluation of a histogram of model residuals and by a plot of the model fitted values against the residuals from the model.

## Results

### Missing Data

In the combined sample of employees from both units, i.e., an intensive care unit (ICU) and paediatric intensive care unit (PICU), 83% of all cases (*N* = 144) had responded to all items in the set of questionnaires, and in total 96% of all data points were complete. Each separate variable had missing values, and data was missing completely at random as assessed with Little’s MCAR (χ^2^ = 44.341, df = 43, *p* = 0.415). Analyses were performed with listwise deletion of cases with missing values. In the longitudinal PICU sample with data from three separate time points, Little’s MCAR test showed data to be missing completely at random (χ^2^ = 118.081, df = 155, *p* = 0.998). LMM analyses of the longitudinal dataset were based on cases with at least one assessment point data for all included variables.

### Participant Characteristics

Means and standard deviations for the self-report questionnaires (total sample, as well as ICU and PICU, respectively) are presented in Table 1. Independent sample *t*-test showed staff from PICU (*M* = 4.48, SD = 0.70) to report significantly higher scores on UWES compared to ICU (*M* = 4.1, SD = 0.75), *t*(136) = 2.80, *p* < 0.01). There were no significant differences between subsamples (ICU, PICU) on age, years of work experience, WAAQ, PSS-10, or GHQ-12.

**TABLE 1:** Mean and standard deviation of each variable presented for total sample (*N* = 144) as well as ICU (*N* = 98) and PICU (*N* = 46) separately.

Variable	Total (ICU + PICU)		ICU		PICU	
	Mean (SD)	N	Mean (SD)	N	Mean (SD)	N
Age	46.0 (10.6)	142	47.7 (9.2)	97	44.2 (11.9)	45
Work experience	19.4 (11.9)	140	20.0 (11.1)	94	18.7 (12.7)	46
*WAAQ*	35.7 (5.5)	143	35.9 (5.5)	97	35.2 (5.5)	46
Men	35.1 (4.4)	14	34.7 (4.3)	13	^a^	1
Women	35.6 (5.5)	128	36.0 (5.5)	83	35.2 (5.6)	39
Physicians	34.9 (4.7)	9	35.0 (5.0)	8	^a^	1
Nurses	35.4 (5.6)	76	35.6 (5.4)	55	35.0 (5.9)	21
Assistant nurses	36.8 (5.7)	47	37.0 (5.8)	32	36.5 (5.8)	15
Miscellaneous	33.3 (3.6)	11	^a^	2	33.6 (3.9)	9
*PSS-10*	13.5 (5.7)	141	13.6 (5.6)	97	13.4 (6.0)	44
Men	12.8 (5.4)	14	13.2 (5.4)	13	^a^	1
Women	13.6 (5.8)	126	13.6 (5.7)	83	13.5 (5.7)	39
Physicians	10.8 (5.8)	9	11.3 (6.0)	8	^a^	1
Nurses	13.9 (6.0)	76	14.1 (5.8)	55	13.5 (6.7)	21
Assistant nurses	13.2 (5.3)	46	13.2 (5.3)	32	13.3 (5.3)	15
Miscellaneous	14.3 (5.5)	10	^a^	2	14.0 (6.0)	9
*UWES Total*	4.2 (0.8)	138	4.1 (0.8)	94	4.5 (0.7)	44
Men	4.3 (0.7)	13	4.3 (0.7)	12	^a^	1
Women	4.2 (0.8)	124	4.1 (0.8)	81	4.5 (0.7)	39
Physicians	4.6 (0.5)	9	4.6 (0.5)	8	^a^	1
Nurses	4.2 (0.8)	74	4.1 (0.8)	54	4.4 (0.8)	21
Assistant nurses	4.2 (0.8)	44	4.1 (0.8)	30	4.5 (0.6)	15
Miscellaneous	4.0 (0.5)	11	^a^	2	4.5 (0.5)	9
*GHQ-12*	8.2 (4.7)	124	8.3 (4.5)	82	8.0 (5.3)	42
Men	6.5 (4.2)	12	6.5 (4.2)	12	^a^	1
Women	8.4 (4.8)	111	8.6 (4.5)	69	7.9 (5.4)	39
Physicians	5.4 (3.7)	8	5.0 (3.8)	7	^a^	1
Nurses	8.9 (5.4)	68	8.7 (4.8)	49	9.2 (6.7)	21
Assistant nurses	7.7 (3.8)	38	8.1 (3.7)	24	7.1 (4.0)	15
Miscellaneous	7.6 (3.3)	10	^a^	2	7.1 (3.2)	9

*WAAQ, work-related acceptance and action questionnaire; PSS-10, perceived stress scale, 10-item version; UWES, Utrecht work engagement scale; GHQ-12, general health questionnaire, 12-item version; ICU, intensive care unit; PICU, pediatric intensive care unit. ^*a*^Results were omitted to maintain confidentiality of individual participants.*

Relationships between age, work experience, and occupational psychological health, as measured by self-report questionnaires, were evaluated using Spearman’s correlation coefficients (see Table 2). Results showed that age was not significantly related to distress, psychological flexibility, or work engagement. Furthermore, results showed that work experience had a positive correlation with WAAQ (*rs* = 0.25, *p* < 0.01), and negative correlations with PSS-10 (*rs* = −0.20, *p* < 0.05) and GHQ-12 (*rs* = −0.20, *p* < 0.05). Work experience did not have a significant correlation with UWES (work engagement).

**TABLE 2:** Bivariate correlation coefficients between self-report questionnaires, and background variables age and work experience.

Variable	1	2	3	4	5	6	7	8
1. Age								
2. Work experience	0.74**							
3. WAAQ	0.06	0.25**						
4. PSS-10	–0.16	−0.20*	−0.37**					
5. UWES Total	0.03	0.07	0.34**	−0.39**				
6. UWES Vigor	0.08	0.16	0.41**	−0.46**	0.86**			
7. UWES Dedication	–0.05	0.01	0.32**	−0.37**	0.90**	0.75**		
8. UWES Absorption	0.06	0.02	0.21*	−0.24**	0.85**	0.54**	0.66**	
9. GHQ-12	–0.12	−0.20*	−0.34**	0.65**	−0.45**	−0.55**	−0.44**	−0.25**

WAAQ, work-related acceptance and action questionnaire; PSS-10, perceived stress scale, 10-item version; UWES, Utrecht work engagement scale; GHQ-12, general health questionnaire, 12-item version; Listwise deletion *N* = 115; *p < 0.05, **p < 0.01.

### Bivariate Relationship Between Psychological Flexibility, Distress, and Work Engagement

Spearman’s correlation coefficients were calculated to evaluate strength and direction of the relationships between psychological flexibility (WAAQ), distress (PSS-10, GHQ-12), and work engagement (UWES Total, UWES Vigor, UWES, Dedication, UWES Absorption) (Table 2). Results showed distress, as measured by PSS-10 (*rs* = −0.39, *p* < 0.001) and GHQ-12 (*rs* = −0.45, *p* < 0.001), to be negatively correlated with work engagement (UWES total). The relationship between psychological flexibility and distress was negative, as shown by the correlation between WAAQ and PSS-10 (*rs* = −0.37, *p* < 0.001) and between WAAQ and GHQ-12 (*rs* = −0.34, *p* < 0.001). Finally, the correlation between psychological flexibility (WAAQ) and work engagement (UWES total) was positive (*rs* = 0.34, *p* < 0.001). Results are summarized in Table 2.

### Ability of Psychological Flexibility to Explain Variance in Work Engagement

Two sets of regression analyses were calculated to assess the amount of variance in UWES (UWES Total, UWES Vigor, UWES Dedication, and UWES Absorption) explained by WAAQ, i.e., with and without controlling for distress (PSS-10 and GHQ-12). Results are summarized in Table 3. Since no background variable (age, years of work experience, profession, gender) were significantly correlated with the dependent variable UWES, they were not included in the regression analyses. However, unit (ICU or PICU) was added as a control variable since there was a significant difference in work engagement (UWES) between units.

**TABLE 3:** Hierarchical multiple regression analyses exploring the ability of the Work-related Acceptance Questionnaire (WAAQ) to explain variance in dependent variables of UWES (UWES total, vigor, dedication, and absorption) in two sets of analyses, model 1 and model 2.

Dependent variable	Model	Step	Predictor variable^b^	*R*^2^	Δ*R*^2^	F change	p (F change)	Standardized beta coefficient^a^		
								Beta	t	p
UWES Total	1	1 2	Unit WAAQ	0.046 0.215	0.046 0.169	6.359 24.426	0.013* 0.001**	−0.242 0.412	−3.126 5.332	0.002** 0.001**
	2	1 2 3	Unit PSS GHQ WAAQ	0.049 0.283 0.347	0.049 0.234 0.064	5.933 18.646 11.059	0.016* 0.001** 0.001**	−0.224 −0.129 −0.275 0.276	−2.941 −1.214 −2.582 3.325	0.004** 0.227 0.011* 0.003**
UWES Vigor	1	1 2	Unit WAAQ	0.040 0.249	0.040 0.208	5.630 36.869	0.019* 0.001**	−0.216 0.457	−2.867 6.072	0.005** 0.001**
	2	1 2 3	Unit PSS GHQ WAAQ	0.024 0.334 0.427	0.024 0.317 0.093	2.849 27.029 18.751	0.094 0.001** 0.001**	−0.166 −0.136 −0.353 0.301	−2.362 −1.395 −3.665 3.934	0.020* 0.166 0.001** 0.001**
UWES Dedication	1	1 2	Unit WAAQ	0.058 0.201	0.058 0.144	8.538 24.819	0.004** 0.001**	−0.269 0.380	−3.528 4.982	0.001** 0.001**
	2	1 2 3	Unit PSS-10 GHQ-12 WAAQ	0.053 0.255 0.307	0.053 0.202 0.052	6.617 15.871 8.701	0.011* 0.001** 0.004**	−0.231 −0.145 −0.240 0.248	−2.976 −1.351 −2.274 2.950	0.004** 0.179 0.025* 0.004**
UWES Absorption	1 2	1 2 1	Unit WAAQ Unit	0.040 0.094 0.047	0.040 0.054 0.047	5.644 8.050 5.813	0.019* 0.005** 0.017*	−0.217 0.233 −0.216	−2.644 2.837 −2.512	0.009** 0.005** 0.013*
		2 3	PSS-10 GHQ-12 WAAQ	0.145 0.164	0.098 0.018	6.597 2.478	0.002** 0.118	−0.185 −0.090 0.147	−1.529 −0.743 1.574	0.129 0.459 0.118

Model 1 evaluates variance in UWES explained by WAAQ while controlling for Unit (ICU, PICU). Model 2 evaluates variance in UWES explained by WAAQ while controlling for unit (ICU, PICU) and distress (PSS-10 and GHQ-12). WAAQ, work-related acceptance and action questionnaire; PSS-10, perceived stress scale, 10-item version; UWES, Utrecht work engagement scale; GHQ-12, general health questionnaire, 12-item version; Regressions were computed with listwise deletion of incomplete cases (*N* = 122–132); ^*a*^Standardized beta coefficients are presented for the whole model (step 2 in model 1, and step 3 in model 2); ^*b*^Units were coded as 0 (PICU) and 1 (ICU); *p < 0.05, **p < 0.01.

In the first set of regression analyses, controlling for the unit, WAAQ explained 16.9% of the variance in UWES Total (β = 0*.412*, *p* < 0.001), 20.8% in Vigor (β = 0*.457*, *p* < 0.001), 14.4% in Dedication (β = 0*.380*, *p* < 0.001), and 5.4% in Absorption (β = 0*.233*, *p* < 0.01). In the second set of analyses, controlling for unit, PSS-10, and GHQ-12, WAAQ explained an additional 6.4% of the variance in UWES Total (β = 0*.276*, *p* < 0.01), 9.3% in Vigor (β = 0*.301, p* < 0.001), and 5.2% in Dedication (β = 0*.248, p* < 0.01). The variance explained by WAAQ in UWES Absorption was non-significant.

### Indirect Effect of Psychological Flexibility on Relationships Between Distress and Work Engagement

Based on theoretical assumptions regarding the relationships between variables, two analyses of indirect effects were calculated. In the first analysis, a significant indirect effect of WAAQ on the relationship between PSS-10 and UWES (ab = −0.0108, 95% CI [−0.0219 – −0.0019]) was seen. The second analyses showed a significant indirect effect of WAAQ on the relationship between GHQ-12 and UWES (ab = −0.0139, 95% CI [−0.0267 – −0.0041]). Expressed in standardized coefficients the effect was −0.0824 in the first analysis (*X* = PSS-10) and −0.0912 in the second analysis (*X* = GHQ-12). Results were significant for subscales Vigor and Dedication and non-significant for subscale Absorption. The direct effect (c’) was significant in all analyses (see Table 4).

**TABLE 4:** Indirect effect of WAAQ in two simple mediation analyses, first on the relationship between PSS-10 and UWES, and second on the relationship between GHQ-12 and UWES.

Mediator: WAAQ						Indirect effect		
*X*	*Y*	*a* path coefficient	*b* path coefficient	Total effect (*c*)	Direct effect (*c’*)	Effect (SE)	CI (95%)	
							LLCI	ULCI
PSS	UWES Tot	−0.359**	0.030**	−0.062**	−0.052**	−0.011* (0.005)	−0.022	−0.002
	UWES Vig	−0.369**	0.042**	−0.079**	−0.064**	−0.016* (0.006)	−0.028	−0.006
	UWES Ded	−0.381**	0.032*	−0.072**	−0.060**	−0.012* (0.006)	−0.025	−0.001
	UWES Abs	−0.370**	0.016	−0.039**	−0.033**	−0.006 (0.006)	−0.018	0.005
GHQ	UWES Tot	−0.418**	0.033**	−0.059**	−0.073**	−0.014* (0.006)	−0.027	−0.004
	UWES Vig	−0.389**	0.048**	−0.094**	−0.075**	−0.019* (0.006)	−0.032	−0.008
	UWES Ded	−0.392**	0.035**	−0.073**	−0.059**	−0.014* (0.007)	−0.028	−0.002
	UWES Abs	−0.418**	0.024	−0.046**	−0.036*	−0.010 (0.006)	−0.024	0.001

WAAQ, work-related acceptance and action questionnaire; PSS, perceived stress scale; UWES, Utrecht work engagement scale; GHQ, general health questionnaire; LLCI/ULCI, Lower Level Confidence Interval/Upper Level Confidence Interval; *p < 0.05, **p < 0.01.

**TABLE 5:** Results of linear mixed model analysis showing estimates of fixed effects of the relationship between time, PSS-10, WAAQ, and dependent variable of UWES.

Estimates of fixed effects ^a^							
Parameter	Estimate	Std. Error	df	t	p	95 % Confidence Interval	
						Lower Bound	Upper Bound
Intercept	4.452	0.077	38.9	59.024	0.001**	4.365	4.674
Time	−0.140	0.046	30.5	−3.043	0.005**	−0.235	−0.046
PSS-10	−0.055	0.011	55.7	−5.095	0.001**	−0.076	−0.033
WAAQ	0.022	0.010	49.0	2.193	0.033*	0.002	0.043

^*a*^Dependent variable: UWES Total; WAAQ, work-related acceptance and action questionnaire; PSS-10, perceived stress scale, 10-item version; *p < 0.05, **p < 0.01.

### Changes in Psychological Flexibility, Distress, and Work Engagement Over Time

The relationships between PSS-10, WAAQ and UWES (outcome) were evaluated with LMM using longitudinal data based on three assessments from a subsample of participants (PICU; *N* = 46). Visual evaluation of model residuals and the plotted model fitted values against model residuals indicated that assumptions of normality were adequately met.

Results showed that there was a significant main linear effect of time on UWES, illustrating a decrease in work engagement across the three assessment points (β = −0.140, *p* < 0.01). Across assessment, results further illustrated that increases in PSS-10 were significantly associated with decreases in UWES (β = −0.055, *p* < 0.001), and that increases in WAAQ were significantly associated with increases in UWES (β = 0.022, *p* < 0.05).

## Discussion

The aim of the present study was to evaluate the relationships between distress, work engagement, and psychological flexibility among intensive care medical staff. Results showed distress (PSS-10 and GHQ-12) to be negatively related to work engagement (UWES). Psychological flexibility (WAAQ) had a positive relation to work engagement (UWES) and a negative relationship with distress (PSS-10, and GHQ-12). Also, hierarchical regression analyses showed that psychological flexibility explained 16.9% of the variance in work engagement. Additionally, when distress (perceived stress and general mental health) was controlled for, WAAQ explained an additional 6.4% of the variance in UWES (total score). Furthermore, there was an indirect effect of psychological flexibility on the relationship between distress (PSS-10, GHQ-12) and work engagement. Finally, results from a linear mixed model analysis showed a significant association between increase in psychological flexibility and increase in work engagement.

Results are consistent with earlier studies on psychological flexibility in occupational health. Corresponding relationships between psychological flexibility, work engagement, and distress was observed in the first study using WAAQ (Bond et al., 2013) as well as in a Spanish sample by Ruiz and Odriozola-González (2014), among Swedish health care professionals (Holmberg et al., 2019), and Chinese oncology nurses (Xu et al., 2018). These studies show similar patterns, with stronger relationships between WAAQ and UWES subscales Vigor and Dedication, and a weaker relationship with the subscale Absorption. Bond et al. (2013) found WAAQ to have incremental validity over personality constructs measured with the Big Five Aspects Scale (BFAS). Results from this study add to those results by also showing WAAQ to have incremental validity over distress, as measured by PSS-10 and GHQ-12.

This is the first study examining indirect effects of WAAQ on the relationship between distress and work engagement. Although tentative, the present findings are consistent with results from intervention studies with a focus on work engagement (Knight et al., 2017; Knight et al., 2019), showing the utility of interventions targeting personal resources/qualities. In addition to intervention research, previous studies have linked work engagement to personal factors within health care (e.g., Fiabane et al., 2013). A recent article addressing psychological flexibility and work engagement among physicians (Solms et al., 2019) also showed psychological flexibility to be associated with work engagement and burnout in residents but not in specialists, which implies differential challenges in work demand that should be addressed in future research.

Interestingly, results indicate similar levels of distress in the present samples compared to normative data on samples from the general population (Cohen and Williamson, 1988; Sconfienza, 1998; Nordin and Nordin, 2013; Lundin et al., 2016). This is in contrasts to previous studies reporting elevated levels of occupational distress among health care professionals in general, and intensive care professionals in particular (Mealer et al., 2009). However, this may in part be clarified by a study by van Mol et al. (2015) that has showed that the prevalence of distress and burnout within intensive care varies extensively within and between units. The large variation should be taken into account when generalizing results from this study, and future research should evaluate if relationship between variables (e.g., psychological flexibility and work engagement) are similar across contexts with different levels of distress.

More research is needed to refine theories and improve staff support and should be built on existing empirical support. For example, future studies should explore the utility of psychological flexibility and work engagement as protective factors of distress, health and work performance (van Mol et al., 2018), as well as the mediating role of perceived stress in the relationship between self-efficacy and work engagement (Pérez-Fuentes et al., 2018).

Limitations and methodological considerations in this study should be taken into account when interpreting the results. Firstly, results based on cross-sectional data cannot be assumed to be consistent with longitudinal data, as shown by e.g., Cain et al. (2018), Maxwell et al. (2011), and O’Laughlin et al. (2018). Consequently, the present results should be evaluated further in future experimental as well as longitudinal studies, which could strengthen the validity of findings regarding relationship between variables. Although the longitudinal analysis with LMM evaluates relationship between variables over time, causality cannot be inferred from this since the present study design lacks experimental qualities as randomization and manipulation of target variable. Furthermore, samples should ideally represent the population to whom the results are subsequently generalized. In this study the PICU subsample is more incomplete (response rate 44%) than the ICU subsample (response rate 78%), and more susceptible to selection bias. In addition, some professions are not sufficiently represented to enable subgroup analyses and explore possible moderation. The present findings should primarily be generalized to populations of nurses and assistant nurses due to the composition of the study sample with a majority of these professions present. Finally, estimation of the size of the indirect effect, to clearer assess the clinical meaningfulness of this effect are difficult. Partially and completely standardized effects can give some information of size (Hayes, 2018), but sizes of indirect effects are often small, and there are yet no standards for reporting effect sizes (MacKinnon et al., 2007). Some have suggested samples of *N* > 500 to reliably evaluate the effect sizes in similar analyses (MacKinnon et al., 2007).

In conclusion, the results of the study preliminary support the utility of psychological flexibility and work engagement to assess and characterize occupational psychological health among intensive care medical staff. Although tentative, the present findings indicate that psychological flexibility may be an addressable and meaningful target for interventions aimed at improving work engagement.

## Data Availability Statement

The datasets for this study will not be made publicly available since the ethical permit does not allow sharing of data. Requests to access the datasets should be directed to johan.holmberg.1@ki.se.

## Ethics Statement

The studies involving human participants were reviewed and approved by the Local Ethical Review Board in Stockholm, Sweden (Registration numbers 2014/42-31/3 and 2015/1881-32/3). The patients/participants provided their written informed consent to participate in this study.

## Author Contributions

JH and RW designed the study. JH performed the collection of data. JH, RW, and MK prepared initial structure of manuscript. JH, MK, and RW chose analytic approach with valuable contributions from LH and L-GÖ. JH and MK performed data analyses. JH, MK, and RW prepared the manuscript with valuable contributions from LH and L-GÖ. All authors approved the final version of manuscript.

## Conflict of Interest

The authors declare that the research was conducted in the absence of any commercial or financial relationships that could be construed as a potential conflict of interest.
